# Importance of Early Recognition and Initiation of Management in Haemophagocytic Lymphohistiocytosis: A Case Report

**DOI:** 10.7759/cureus.97645

**Published:** 2025-11-24

**Authors:** Sreemallika Ramireddy, Sarah Jones

**Affiliations:** 1 General Medicine, Aneurin Bevan University Health Board, Newport, GBR

**Keywords:** acute liver failure (alf), anakinra treatment, cmv -induced hlh, deranged coagulopathy, ebv induced hlh, elevated ferritin level, haemophagocytic lymphohistiocytosis, h score, “pancytopenia”, sepsis-like syndrome

## Abstract

We present the case of an 18-year-old previously healthy male who was admitted with severe abdominal pain and subsequently found to have concurrent Epstein-Barr virus (EBV) and cytomegalovirus (CMV) infections. Due to concern over potential deterioration, he was transferred to critical care, where progressive cytopenias and deranged liver function tests were noted. The combination of acute liver failure, pancytopenia, hyperferritinaemia, and coagulopathy raised suspicion for haemophagocytic lymphohistiocytosis (HLH). This was supported by bone marrow aspirate findings demonstrating megakaryocytic proliferation and elevated soluble CD25 levels on further testing performed at the Great Ormond Street Hospital (GOSH).

High-dose methylprednisolone and anakinra were initiated with subsequent clinical and biochemical improvement. Targeted antiviral therapy with ganciclovir and rituximab was commenced for CMV and EBV, respectively. The patient’s haematological parameters and liver function were normalised, and he was discharged on a tapering regimen of prednisolone and anakinra, alongside oral valganciclovir.

This case highlights the importance of early recognition of HLH in the context of viral coinfection, particularly EBV and CMV. Prompt multidisciplinary involvement and initiation of immunosuppressive and antiviral therapy are crucial to improving outcomes in this potentially fatal condition.

## Introduction

Haemophagocytic lymphohistiocytosis (HLH) is a potentially fatal hyperinflammatory syndrome defined by a disproportionate immune response involving natural killer cells and T cells [1-3]. Organ failure, leading to intensive care admissions and death are potential consequence of missed diagnosis [1,4], highlighting the importance of early recognition and initiation of treatment. There are two categories of HLH: primary, which usually presents in childhood with a familial genetic component, and secondary, which arises following an acute immune response, such as in malignancy, sepsis, or immunological conditions, more common in the adult population [1,2,4]. 

Diagnosis is often difficult due to the presentation being similar to that of haematological malignancies or infections causing sepsis [1,4]. The diagnosis of HLH was previously considered if the patient met the HLH-94 or the subsequent HLH-2004 criteria [3]. However, the probability of an HLH diagnosis can now be performed using the HScore, which looks for physical signs, for example, splenomegaly or pyrexia, presence of biochemical markers, such as cytopenias, and the presence of haemophagocytosis on bone marrow aspirate [4,5]. Once a diagnosis is made, treatment can be initiated with the aid of the multidisciplinary team (MDT), involving haematologists and, if appropriate, microbiology specialists. 

Treatment of HLH involves management that would be counterproductive and dangerous in cases that present similarly, for example, in severe sepsis, as the mainstay is immunosuppression and cytotoxic medications [1]. In this case, treatment involved high-dose corticosteroids for immunosuppression and anakinra, leading to significant clinical improvement. 

We present the case of an 18-year-old previously healthy male who was admitted with severe abdominal pain and subsequently found to have concurrent Epstein-Barr virus (EBV) and cytomegalovirus (CMV) infections, leading to a diagnosis of HLH.

## Case presentation

Over the course of one week (from 10th to 16th August), an 18-year-old male presented to the Emergency Department (ED) twice with a two-day history of sore throat, reduced oral intake, and a sensation of facial and neck swelling. He had no significant past medical history and no known consanguinity. On both occasions, he was reviewed by the Ear, Nose, and Throat (ENT) team, received intravenous dexamethasone, fluids, and analgesia, and was discharged once able to tolerate oral intake with a prescription for oral phenoxymethylpenicillin. The patient's first admission lasted 10 hours and 43 minutes, with a working diagnosis of presumed tonsillitis. Examination findings were documented as “bilateral lymphadenopathy, grade 3 tonsils with exudate, full neck range of motion, no trismus.”

Due to persisting symptoms and being unable to maintain good oral intake, the patient returned to the ED 14 hours later. During this second admission (lasting five days, from 11th to 16th August), a glandular fever screen returned positive. Blood tests showed an elevated white cell count of 26.5 × 10⁹/L, lymphocytes 11.4 × 10⁹/L, and atypical lymphocytes on blood film. Immunophenotyping was negative for lymphoma. 

Five days after the second discharge, the patient re-presented to the ED with sudden-onset severe abdominal pain, hypotension, and tachycardia. Examination revealed splenomegaly, raising the suspicion of spontaneous splenic rupture. A CT abdomen and pelvis (CTAP) demonstrated splenomegaly without evidence of rupture or haematoma, mild hepatomegaly, reactive gallbladder wall oedema, and mesenteric invagination into the small bowel suggestive of intussusception. He was taken for emergency diagnostic laparoscopy on the 21st of August, which revealed no evidence of current or recent intussusception. Intraoperatively, the surgeons noted a diffusely “oozing” omentum, deranged coagulation on rotational thromboelastometry (ROTEM), and a markedly enlarged liver and spleen. He was transferred to the Intensive Therapy Unit (ITU) on the 22nd of August, for monitoring in view of possible acute liver failure and anticipated post-operative deterioration. 

On the day of ITU admission, the patient developed high fever, anaemia, and thrombocytopenia. A calculated HLH probability score indicated >99% likelihood of HLH (Table 1). Following haematology review, high-dose methylprednisolone and anakinra were commenced. The patient’s liver function improved with supportive management. Virology studies revealed high levels of both CMV and EBV PCR positivity, suggesting either CMV reactivation secondary to immunosuppression or a dual infection. On virology advice, ganciclovir was initiated for CMV, and rituximab for EBV. 

**Table 1:** HScore criteria met by our patient, scoring a probability of >99% of HLH Source: Ref. [6]. Hb, haemoglobin; AST, aspartate aminotransferase; HLH, haemophagocytic lymphohistiocytosis.

HScore Criteria	Our Patient Data
Underlying Immunosuppression	Yes
Fever	No Fever >40°C
Organomegaly	Splenomegaly and Hepatomegaly Present
Number of Cytopenias - Defined as Hb <90 g/L and/or Platelets <100 × 10^9^/L and/or White Blood Cells <5.0 x 10⁹/L	Two Lineages (Hb of 81 g/L, Platelets of 52 x 10^9^/L)
Ferritin (Reference Range: 15-300 µg/mL)	Highest Recorded – 9347 µg/mL
Triglycerides (Reference Range: <2 mmol/L)	9.5 mmol/L
Fibrinogen (Reference Range: 2-4 g/L)	0.6 g/L
AST (Reference Range: <41 U/L)	1026 U/L
Haemophagocytosis on Bone Marrow Aspirate	Present

**Figure 1:**
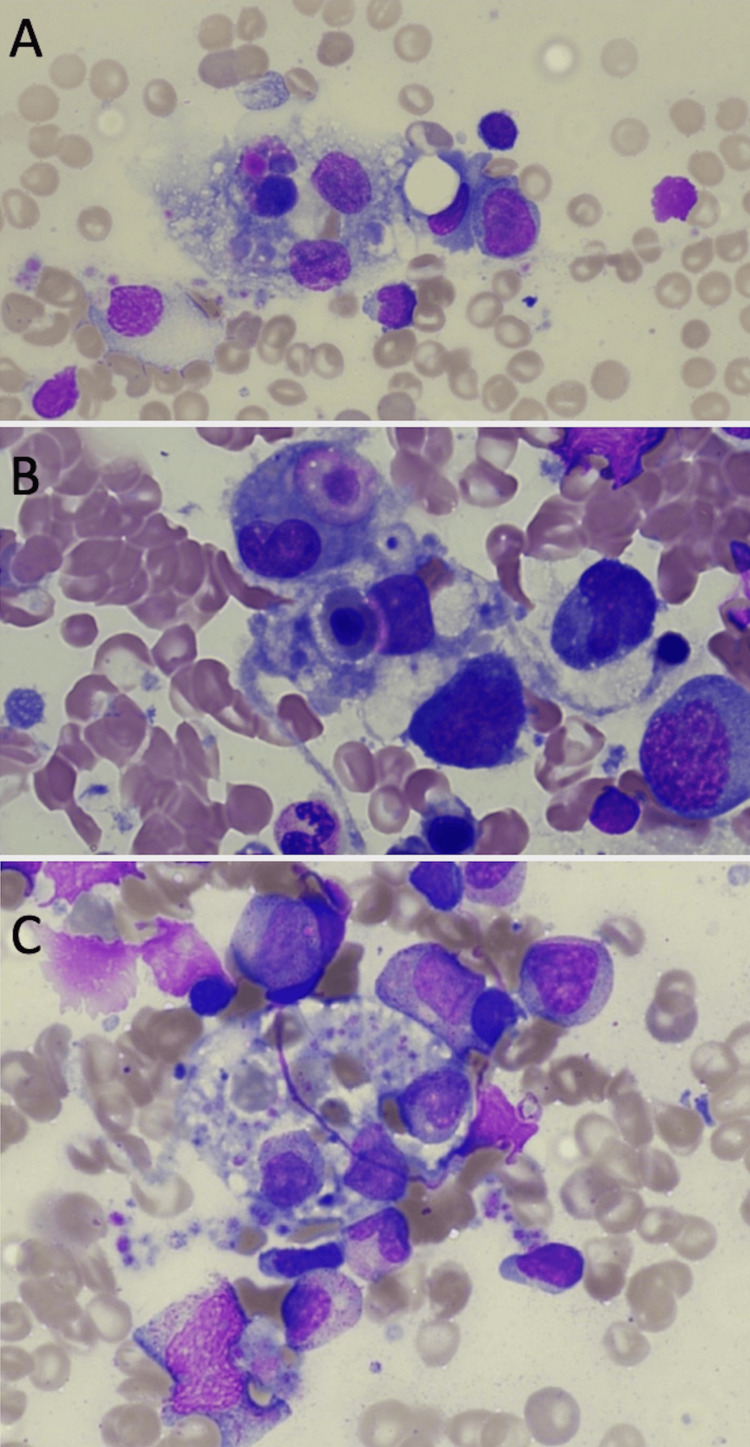
Histopathology images from the patient's bone marrow biopsy specimens Bone marrow aspiration was performed on 28th August. The aspirate was stained with May-Grunwald Giemsa (MGG). Original magnification used, x50 with immersion oil. Increased histiocytic activity is seen in patients with HLH, with histiocytes ingesting and destroying bone marrow and blood cells. This is called haemophagocytosis and is demonstrated in these images (A-C).

Bone marrow biopsy demonstrated increased histiocytic activity with evidence of haemophagocytosis, consistent with HLH (Figure 1). The case was discussed at the national HLH MDT meeting (held at University College London Hospital), and functional studies were sent to GOSH, alongside an R15 genetic panel to the local genetics team. Genetic testing yielded a negative result, with no detectable variants to indicate the presence of a hereditary disorder.

At his most recent outpatient review (on the 1st of October), HLH markers had normalised. The patient is being gradually weaned off prednisolone and anakinra. He was discharged on oral Valganciclovir, which is being continued as CMV remains detectable (2564 copies/mL). The virology team will be consulted regarding the duration of antiviral therapy.

## Discussion

Distinguishing HLH from other causes of systemic inflammation, such as sepsis or severe viral infection, remains challenging, often delaying diagnosis and treatment initiation. EBV is the most frequently implicated viral trigger of HLH, with pathogenesis linked to excessive activation of infected cytotoxic T cells and macrophages. CMV-related HLH is less common and typically occurs in immunocompromised individuals [7,8]. This unusual combination of EBV and CMV infection in an immunocompetent individual highlights how concurrent viral immune activation can overwhelm normal regulatory mechanisms and perhaps precipitate HLH. 

In this case, the combination of progressive cytopenias, hyperferritinaemia, and coagulopathy prompted calculation of the HScore, which indicated a >99% probability of HLH. The diagnosis was further supported by elevated soluble CD25 levels and characteristic bone marrow findings. This highlights the importance of maintaining a high index of suspicion and applying standardised diagnostic tools when laboratory features are disproportionate to the initial presumed infection. 

Management of infection-associated HLH requires prompt initiation of immunosuppressive therapy to mitigate the hyperinflammatory response, while concurrently addressing the underlying trigger [9,10]. In this case, high-dose corticosteroids and anakinra led to rapid clinical improvement, with concurrent antiviral therapy (ganciclovir and rituximab) targeting CMV and EBV replication (Table 2). 

**Table 2:** Improvement in blood findings after initiation of treatment – anakinra, corticosteroids, rituximab and valganciclovir on 23rd August Reference ranges used are those provided by the pathology laboratory at Grange University Hospital, part of Aneurin Bevan University Health Board, South Wales, United Kingdom.* * EBV, Epstein-Barr virus; PCR, polymerase chain reaction; CMV, cytomegalovirus; ALT, alanine transaminase; AST, aspartate aminotransferase; ALP, alkaline phosphatase.

Laboratory Tests	Reference Range	23rd Aug	04th Sep	17th Sep	30th Sep
EBV PCR (IU/mL)	<320	698,880	<1500	<1500	Negative
CMV PCR (IU/mL)	<305	107,883	91,617	5436	1615
Ferritin (µg/mL)	15-300	6859	1509	202	107
Triglycerides (mmol/L)	<2	5.9	9.9	3.1	2.7
ALT (U/L)	<41	576	490	183	96
AST (U/L)	<41	1026	441	Not tested	42
ALP (U/L)	59-294	437	941	379	166

Long-term follow-up remains essential in HLH, even in apparently idiopathic or infection-triggered cases. Relapse can occur months after apparent remission, particularly if an underlying genetic predisposition is present. Functional studies and a targeted gene panel (R15) were therefore performed to exclude primary or familial HLH. The absence of recurrent symptoms and normalisation of HLH markers are reassuring in this patient; however, continued surveillance remains important, given the risk of HLH recurrence.

Early multidisciplinary input contributed significantly to this patient’s favourable outcome. As the HScore was first calculated during his third admission, it is reasonable to consider whether earlier application of standardised assessment tools may have expedited diagnosis and reduced the overall length of hospitalisation.

## Conclusions

In conclusion, this case represents an unusual example of HLH secondary to concurrent EBV and CMV infection in an immunocompetent young adult. It highlights the diagnostic complexity of HLH, the importance of prompt immunosuppressive and antiviral therapy, and the critical role of multidisciplinary management. Increasing awareness of HLH among frontline clinicians may facilitate earlier recognition and intervention, ultimately improving survival in this highly treatable but potentially fatal syndrome. Detailing cases where the underlying driver remains unclear contributes to the broader evidence base needed to better characterise atypical or multifactorial HLH presentations.
